# *Meconopsis biluoensis* (Papaveraceae), a new species revealed by population‐level investigation

**DOI:** 10.1002/ece3.11323

**Published:** 2024-04-29

**Authors:** Hao Wang, Tian‐Yi Yu, Yi Fan, Lai Wei

**Affiliations:** ^1^ College of Life Sciences Beijing Normal University Beijing China; ^2^ Ministry of Education Key Laboratory of Biodiversity and Ecological Engineering Beijing Normal University Beijing China; ^3^ Herbarium Royal Botanical Gardens, Kew Surrey UK; ^4^ Yunnan Natural and Cultural Heritage Conservation Council Kunming China

**Keywords:** endemic species, *Meconopsis biluoensis*, morphology, Papaveraceae, phylogeny

## Abstract

*Meconopsis biluoensis*, a new species of Papaveraceae in an alpine meadow from Yunnan, Southwest China, is described and illustrated. Morphologically, it resembles *Meconopsis georgei*, while it is distinct in acaulescent and hispid with clearly expanded bases on the leaves. A genus‐level molecular phylogenetic analysis supported the closest relationship between *M. biluoensis* and *M. georgei*. In a finer population‐level molecular phylogenetic analysis using ribosomal DNA (rDNA) and the chloroplast genome, individuals from *M. biluoensis* and *M. georgei* were clearly separated, and the extremely short branch length indicated that the two species had a very short differentiation time. The species has currently been assessed as “endangered” (EN) due to its small‐sized population and narrow distribution following the IUCN categories and criteria.

## Graphical abstract

A beautiful and rare plant, *Meconopsis biluoensis*, was discovered through population‐level studies. The new species belongs to Section Aculeatae and differentiates from its most related species *Meconopsis georgei* by inflorescence and indumenta.

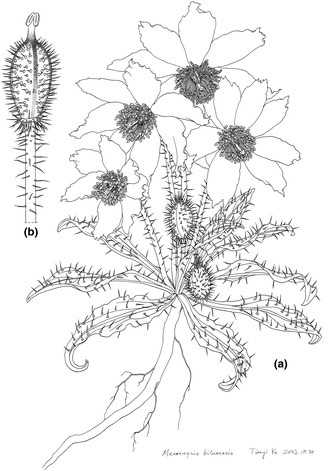

## 1 INTRODUCTION

*Meconopsis* Vig. is the second largest genus of Papaveraceae, with all species found in the Sino‐Himalayan region (Egan, 2011). Because of its extremely beautiful flowers, the genus has been the flagship example of the regional flora since the mid‐19th century (Grey‐Wilson, 2014; Mueggler, 2011). In the first decade of this century, many new species were published (An et al., 2009; Egan, 2011; Grey‐Wilson, 2006; Ohba et al., 2009; Yoshida et al., 2010, 2011), increasing the total number of *Meconopsis* from approximately 50 (Mabberley, 2008) to approximately 80 (Grey‐Wilson, 2014). However, because of the high level of interest and relatively detailed research, the number of new species published has begun to decline in recent years. On the other hand, due to reassessment of the species concept and vague criteria for the classification of species, the delineation between closely related species in *Meconopsis* is very chaotic (Xie et al., 2014). This situation makes the publication of new species in *Meconopsis* more difficult.

In 2018, we discovered a distinctive *Meconopsis* species during a botanical exploration in Nanjieluo, Badi village, Weixi County, Yunnan Province. After comparing a single nuclear ITS marker, we believed the species should be treated as *Meconopsis georgei* Taylor, a species that had not been found for over 80 years, ever since it had been published in 1934 (Wei et al., 2019). At the time, we realized that there were some differences between the species we found in Nanjieluo and the protologue of *M. georgei*, but when consulting the whole genus, these differences did not exceed the range of infraspecific variation. For the next 5 years, we have continued to monitor the species in Nanjieluo and conducted a botanical exploration in Fuchuan Mountain this year, the type locality of *M. georgei*. In Fuchuan Mountain, we collected individuals who were nearly the same as those in the protologue of *M. georgei* morphologically. After carefully comparing the chloroplast genome and nuclear gene rDNA at the population level, we believe that there are stable differences between the individuals in Nanjieluo and Fuchuan Mountain, both morphologically and genomically. Therefore, the individuals found in Nanjieluo should be a new species in *Meconopsis*.

## 2 MATERIALS AND METHODS

### 2.1 Sampling, DNA extraction, sequencing, and assembly

The sequences utilized in this study encompass both novel sequences and previously published sequences. Leaf materials of 26 individuals representing seven species (13 *Meconopsis biluoensis*, 5 *M. georgei*, 3 *Meconopsis castanea*, and 5 other *Meconopsis* species, including *Meconopsis exilis*, *Meconopsis impedita*, *Meconopsis sulfurea*, *Meconopsis bijingensis*, and *Meconopsis pratti*) were obtained from Yunnan, China.

Total genomic DNA was extracted and purified using the Plant Genomic DNA Rapid Extraction kit (Tiangen Corporation, Beijing, China). The quality of the DNA was evaluated using a NanoDrop spectrophotometer (Thermo Scientific, Carlsbad, CA, USA), while the integrity of the DNA was assessed through electrophoresis on a 1% (w/v) agarose gel. A DNA library was prepared using the NEB Next Ultra DNA Library Prep Kit (Illumina Inc., San Diego, CA, USA). The DNA libraries were then sequenced on an Illumina NovaSeq 6000 (Illumina Inc.) platform to generate 150‐bp paired‐end reads, with approximately 5 GB of data for each sample. Low‐quality reads from raw data were filtered using fastp v 0.23.1 to remove sequencing adaptors and low‐quality bases (Chen et al., 2018). The filtered reads were assembled using the GetOrganelle v1.7.7.0 program for plastomes and nrDNA (Jin et al., 2020). The chloroplast genome sequences were initially annotated using Geneious Prime by referring to the cp genome sequence of *Meconopsis punicea* (MW233592.1) and subsequently refined through manual calibration based on the open reading frame. The NCBI database accession numbers of the new sequences (cp genomes, nr DNA) in this study and other species for molecular phylogenetic analysis are shown in Table A1.

### 2.2 Phylogenetic analysis

Phylogenetic analysis was inferred using maximum likelihood (ML) and Bayesian inference (BI) methods. Three datasets were generated for phylogenetic analyses as follows: (1) 23 whole plastomes; (2) 50 maturase K (*mat*K) genes, complete cds; and (3) 22 whole nrDNA sequences. *Papaver alpinum* were chosen as outgroups to root the *mat*K tree (Xiao & Simpson, 2017). Sequences were aligned using MAFFT v.7.215 (Katoh et al., 2019). The ML analyses were performed using IQ‐TREE v 2.0.3 with 1000 bootstrap replicates (Nguyen et al., 2015). The best‐fitting model was determined using ModelFinder. Bayesian inference (BI) analysis was performed with MrBayes 3.2.7a (Ronquist & Huelsenbeck, 2003), employing two parallel runs for a total of 10 million generations and sampling every 5000 generations; the initial 25% of trees were discarded as burn‐in. Posterior probabilities (PP) were estimated based on a majority‐rule consensus tree with a threshold of 50%. The effective sample size (>200) was assessed using Tracer v1.7 (Rambaut et al., 2018). Visualization of the trees was conducted using Figtree V.1.4.4 (Rambaut, 2014).

## 3 RESULTS

**FIGURE 1:**
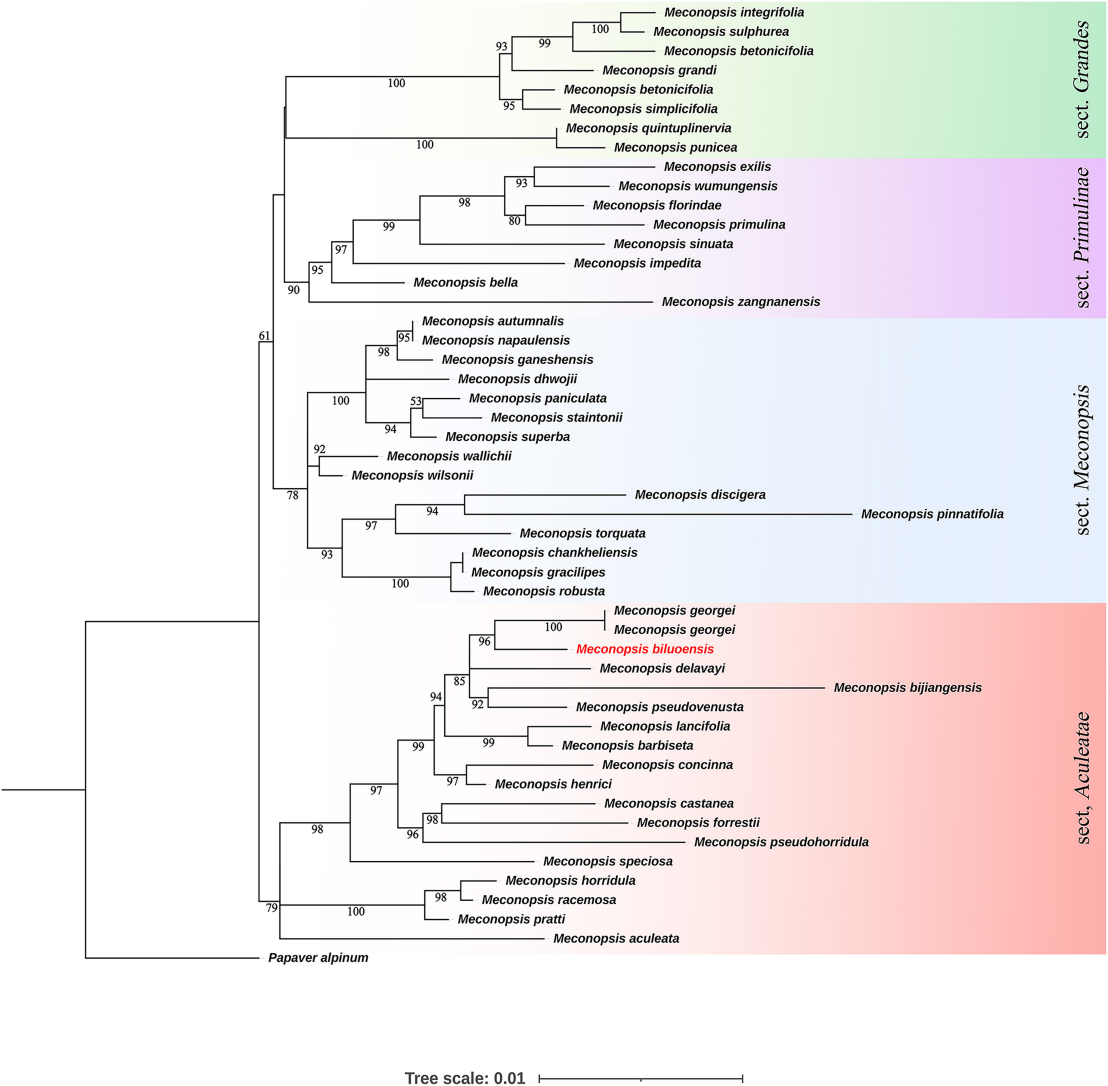
ML tree inferred from *mat*K sequence data. Values below branches are ML bootstrap percentages. *Meconopsis biluoensis* is shown in red.

In the genus‐level molecular phylogenetic analysis using the single chloroplast gene *mat*K, our result was highly congruent with that of earlier studies (Xiao & Simpson, 2017). In the maximum likelihood tree, four clades were formed. The inferred new species *M. biluoensis* was embedded in sect *Aculeate* according to Xiao and Simpson (2017), together with *M. georgei*. The two species formed a monophyletic group in clade *Aculeate* (Figure 1).

**FIGURE 2:**
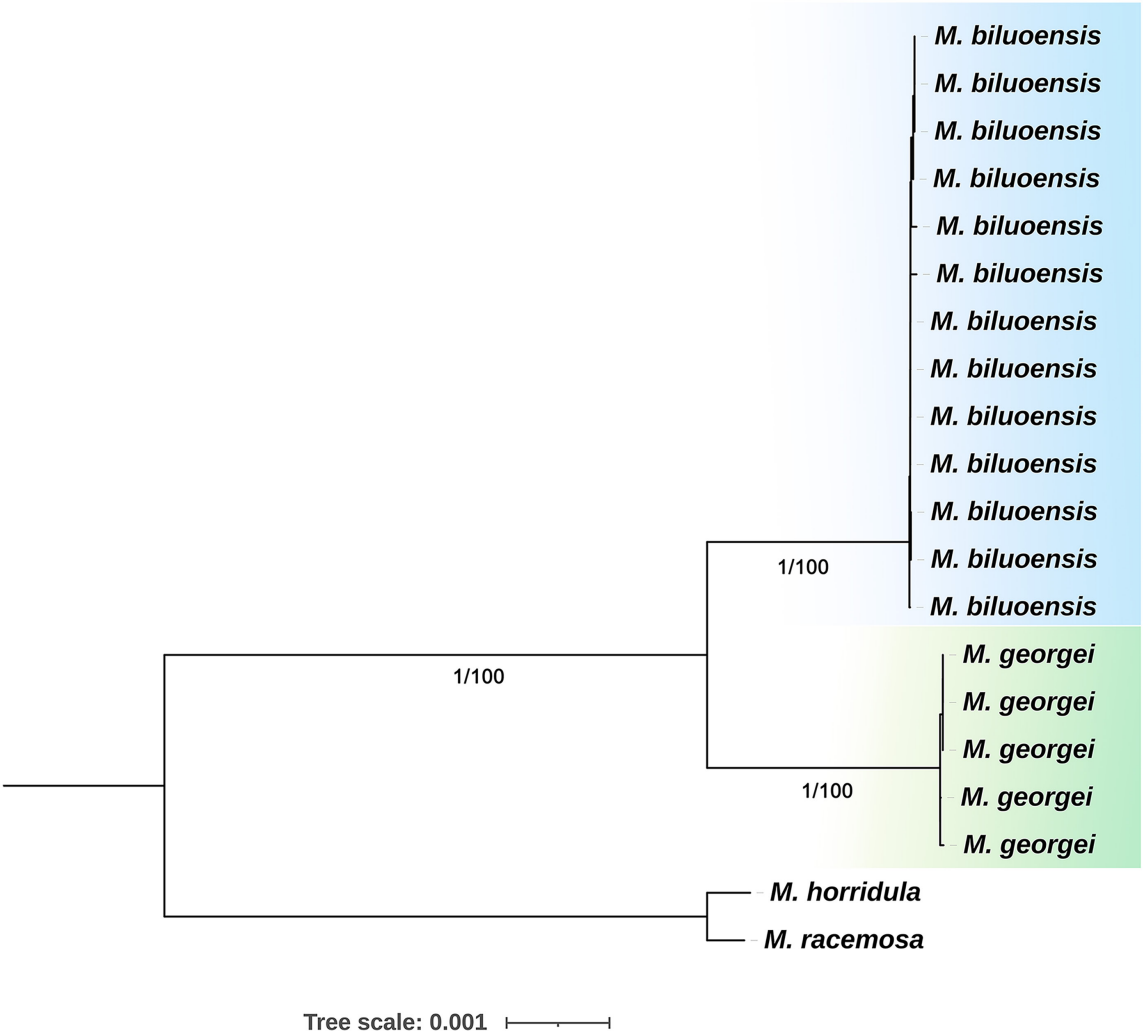
Population level tree inferred from cp genome focusing only *Meconopsis georgei* and *Meconopsis biluoensis*. Values below branches are ML bootstrap percentages and Bayesian posterior probabilities.

**FIGURE 3:**
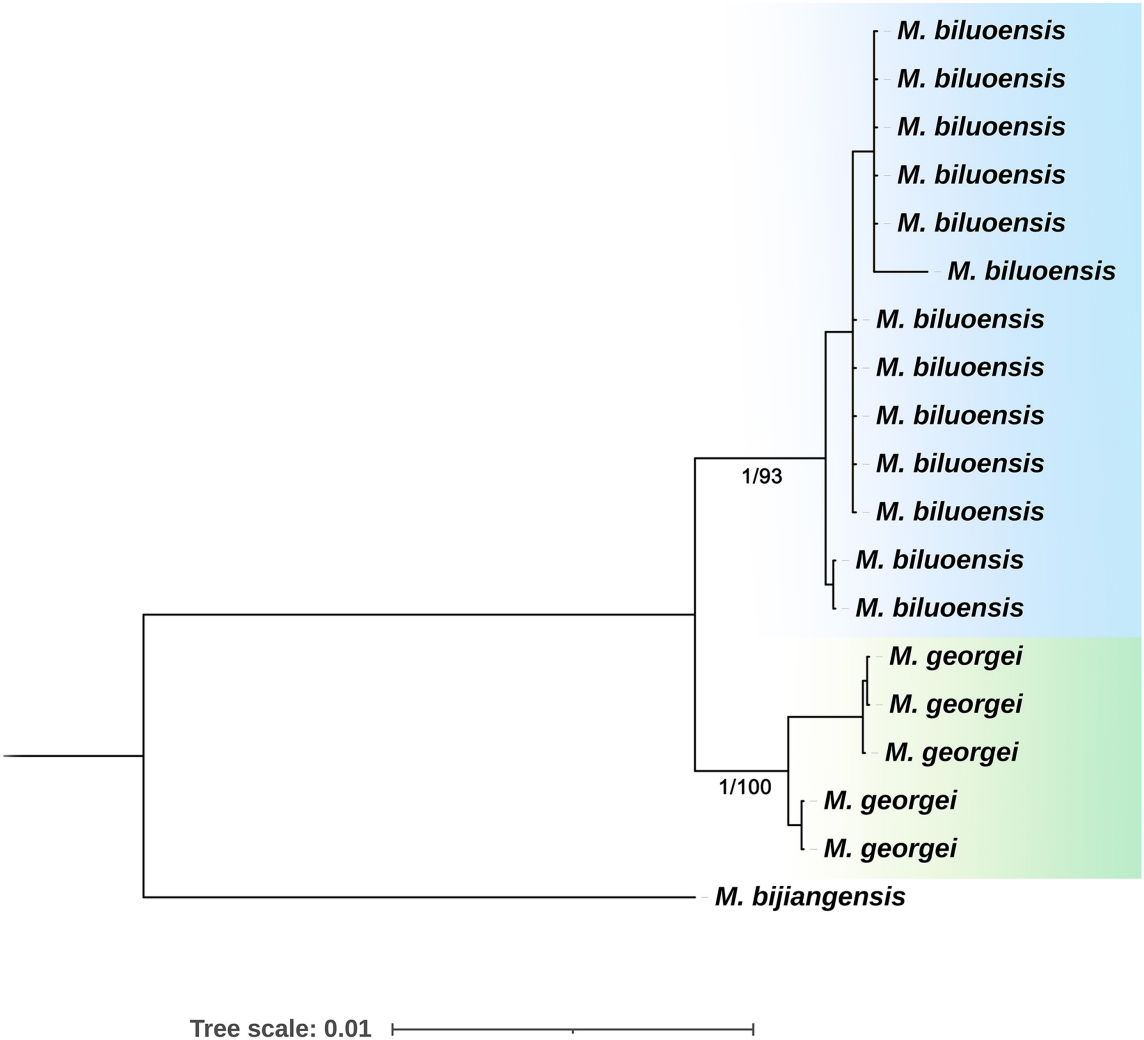
Population level tree inferred from nrDNA focusing only *Meconopsis georgei* and *Meconopsis biluoensis*. Values below branches are ML bootstrap percentages and Bayesian posterior probabilities.

To further investigate the genetic differentiation between *M. biluoensis* and *M. georgei*, we carried out a more detailed molecular phylogenetic study using the chloroplast genome and nuclear rDNA at the population level. In this analysis, *M. biluoensis* was represented by five individuals, and *M. georgei* was represented by 13 individuals. Both the chloroplast genome (Figure 2) and nrDNA tree (Figure 3) of the ML and BI methods show nearly the same topology. Individuals from different species were grouped together separately with the highest support.

## 4 DISCUSSION

**FIGURE 4:**
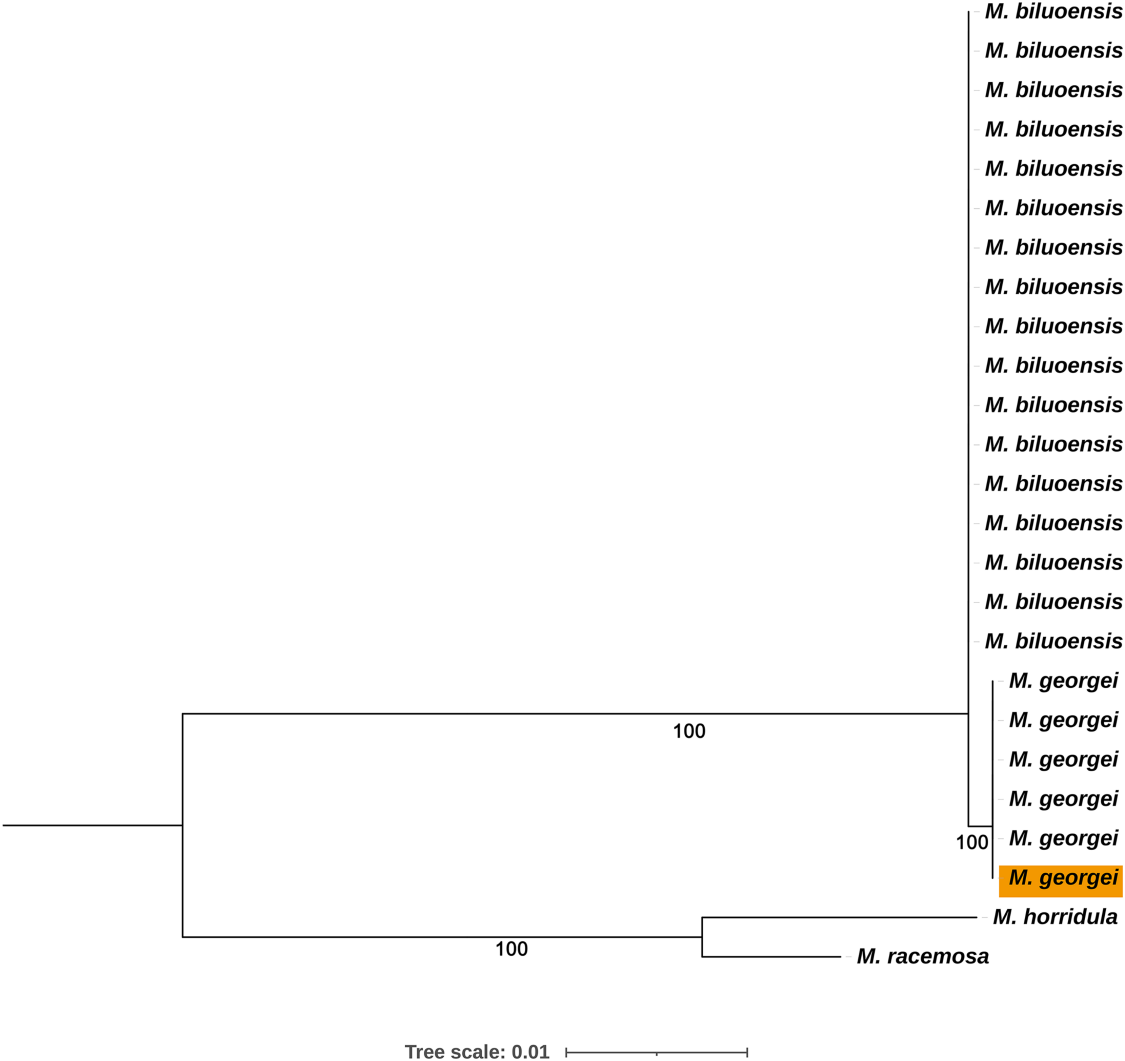
Population‐level tree inferred from ITS focusing only *Meconopsis georgei* and *Meconopsis biluoensis*. Values below branches are Bayesian posterior probabilities. The orange *M. georgei* represents the sequence from the same gathering as the type specimen of *M. georgei* which already exists in the GenBank.

The genus‐level molecular phylogenetic tree shows that *M. georgei* and *M. biluoensis* formed a monophyletic group and were embedded in section *Aculeatea* according to Xiao's classification (Xiao & Simpson, 2017). In a further population‐level molecular phylogenetic analysis, both the chloroplast genome and nuclear rDNA supported the monophyly of the two species. Our previous results showed that it was not possible to differentiate *M. biluoensis* from *M. georgei* using ITS (Wei et al., 2019). With the number of samples increasing, we conducted another molecular phylogenetic analysis using ITS (Figure 4). In the ITS tree, *M. georgei* forms a monophyletic group, together with a sequence from GenBank (No. JX078989.1), which was amplified from the same gathering as the type specimen of *M. georgei* collected by Georgei Forrest. Due to poor resolution caused by insufficient information sites, *M. biluoensis* is paraphyletic apart from *M. georgei*. However, as the sequence length and information sites increase, the monophyly of *M. biluoensis* is equally well demonstrated at the biparental genetic marker rDNA sequence (Figure 3). It is worth noting that the branch lengths of *M. biluoensis* and *M. georgei* are extremely short, indicating that the differentiation time of these two species is very short. This result may explain the subtle morphological differences between the two species.

*Meconopsis* is an herbaceous genus native to high‐altitude habitats across the Himalayas and adjacent plateau and mountain areas. The Himalo‐Tibetan region is the best‐known region in the world to be undergoing orogenesis (Van Hinsbergen et al., 2012). Many studies suggest that mountain uplift can drive the evolution of new species and the rapid differentiation of species (Hughes & Eastwood, 2006; Meng et al., 2007; Winkworth et al., 2005). *Meconopsis* may be undergoing these processes. During speciation and rapid differentiation, differences between species may have developed gradually, accompanied by hybridization (De Queiroz, 2007), making morphological features within genera more complex than we can imagine. Therefore, we must be very careful when addressing the nuances between species in Meconopsis.

**FIGURE 5:**
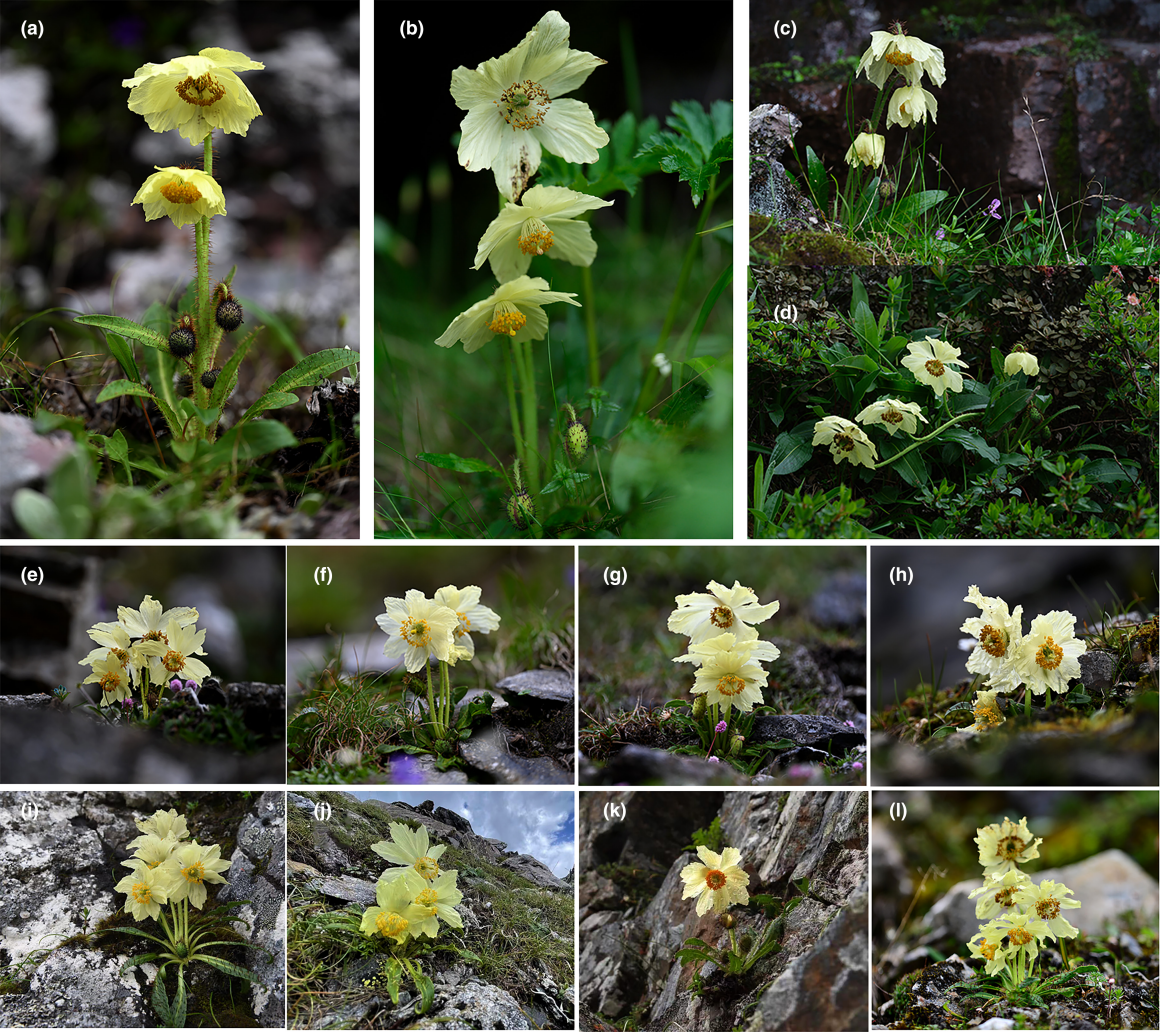
Individuals from *Meconopsis georgei* and *Meconopsis biluoensis*, show the stable differences in inflorescences. (a–d) *M. georgei*; (e–l) *M. biluoensis*.

The main difference between *M. biluoensis* and *M. georgei* is the type of inflorescence. Scapes versus racemose is a vital difference discussed by many authors in their monograph of *Meconopsis* to distinguish sections and series (Grey‐Wilson, 2014; Taylor & Cox, 1934; Wu et al., 1999; Zhang & Grey‐Wilson, 2008). Flowers of *M. biluoensis* are born on leafless scapes, while the flowers of *M. georgei* are arranged into a racemose. We noticed this difference in 2019. When consulting the type specimens, we noticed that the inflorescence was not a typical racemose, and all flowers were loosely arranged on long, slender pedicels along the elongated stem. It is easy to imagine that when some individuals grow shorter, lax racemose can change into leafless scapes (Wei et al., 2019). This situation occurs in other species in *Meconopsis*, such as *M. horridula* and *M. lancifolia*. However, after 5 years of observation, we found that the difference in inflorescence was fairly stable between the two species (during the 5‐year monitoring process, we recorded over 50 flowering plants of *M. biluoensis*, and all individuals possessed leafless scapes; in contrast, all individuals of *M. georgei* we saw this year possessed lax racemose [Figure 5]). Therefore, the type of inflorescence could be an important diagnostic feature between *M. biluoensis* and *M. georgei*.

**FIGURE 6:**
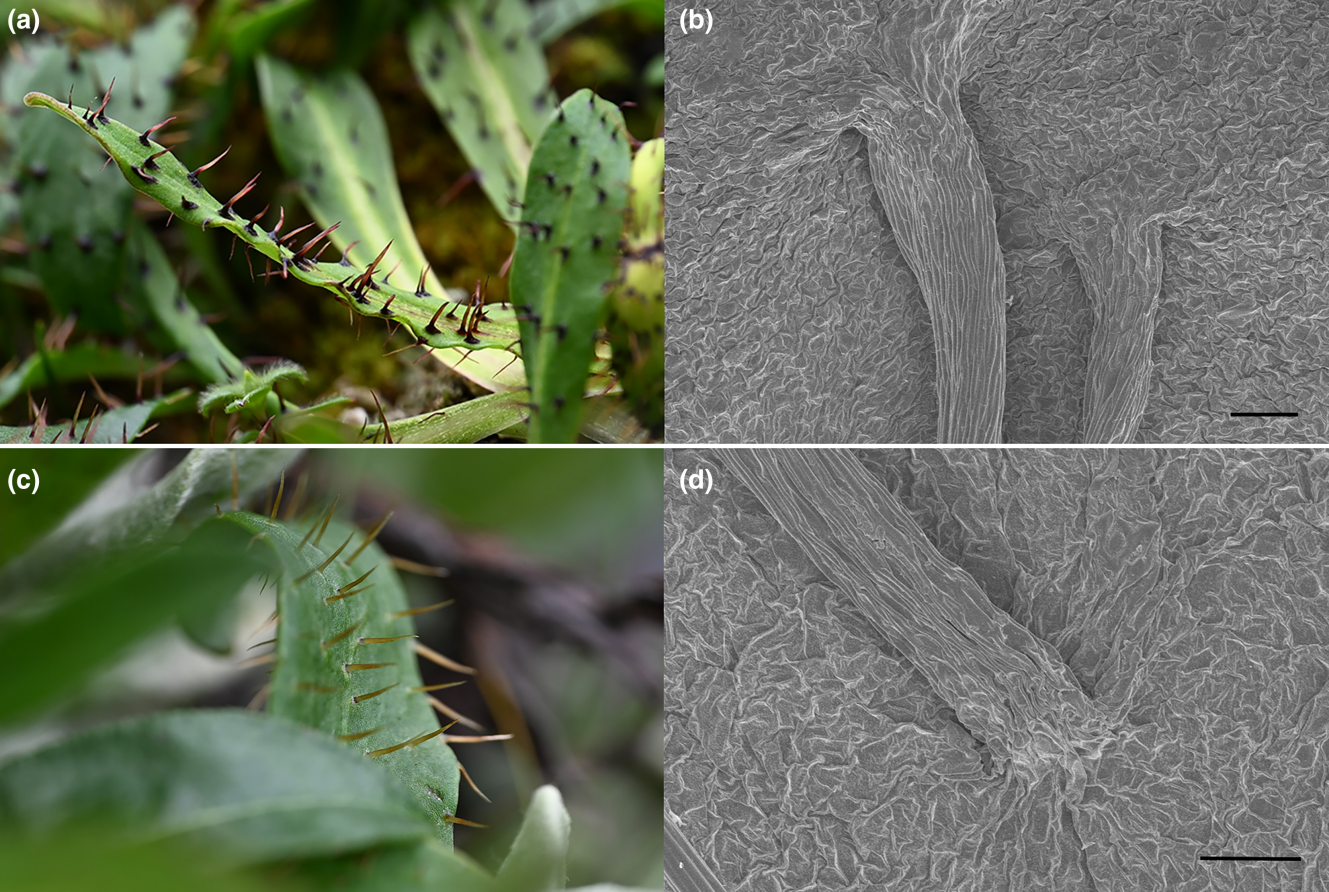
Difference of indumenta between *Meconopsis biluoensis* and *Meconopsis georgei*. (a) Leaves of *M. biluoensis*; (b) hispid of *M. biluoensis* under SEM, showing expanded bases; (c) leaves of *M. georgei*; (d) hispid of *M. georgei* under SEM, showing the hispid without expanded bases. Bars = 100 μm.

Another difference is the indumenta, especially on the leaves. In *M. biluoensis*, the hispid have conspicuous expanded bases, while in *M. georgei*, hispid scarcely expand at the base, which was also mentioned by Grey‐Wilson in his monograph on *Meconopsis* (Grey‐Wilson, 2014). It is worth noting that when only comparing specimens, the difference becomes less insignificant because the expanded base of the hairs collapsed when losing moisture during the process of making specimens. That is the reason we did not take it as a diagnostic feature before. However, when we take a closer observation of plants in their living state, the difference is obvious and stable at the population level. At the same time, we performed a simple scanning electron microscopy observation. In the case of dehydration, whether the base is expanded is still very obvious between the two species (Figure 6). In summary, combining molecular phylogeny and morphological surveys at the population level, we believe *M. biluoensis* is a separate species with a very short history of differentiation.

### 4.1 Taxonomic treatment

**FIGURE 7:**
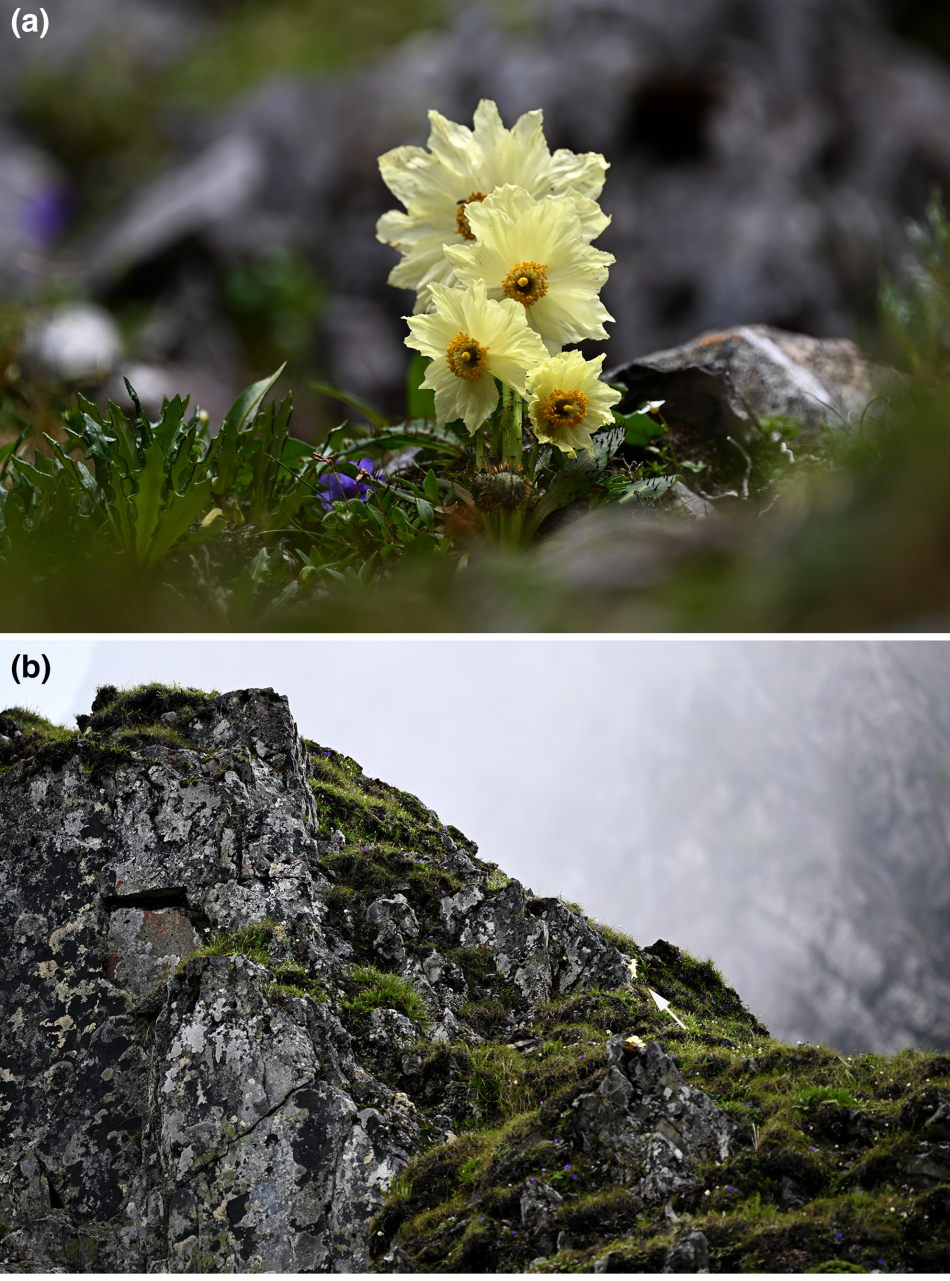
*Meconopsis biluoensis* and its growing habit. The arrow indicate *M. biluoensis* in the living stage.

**FIGURE 8:**
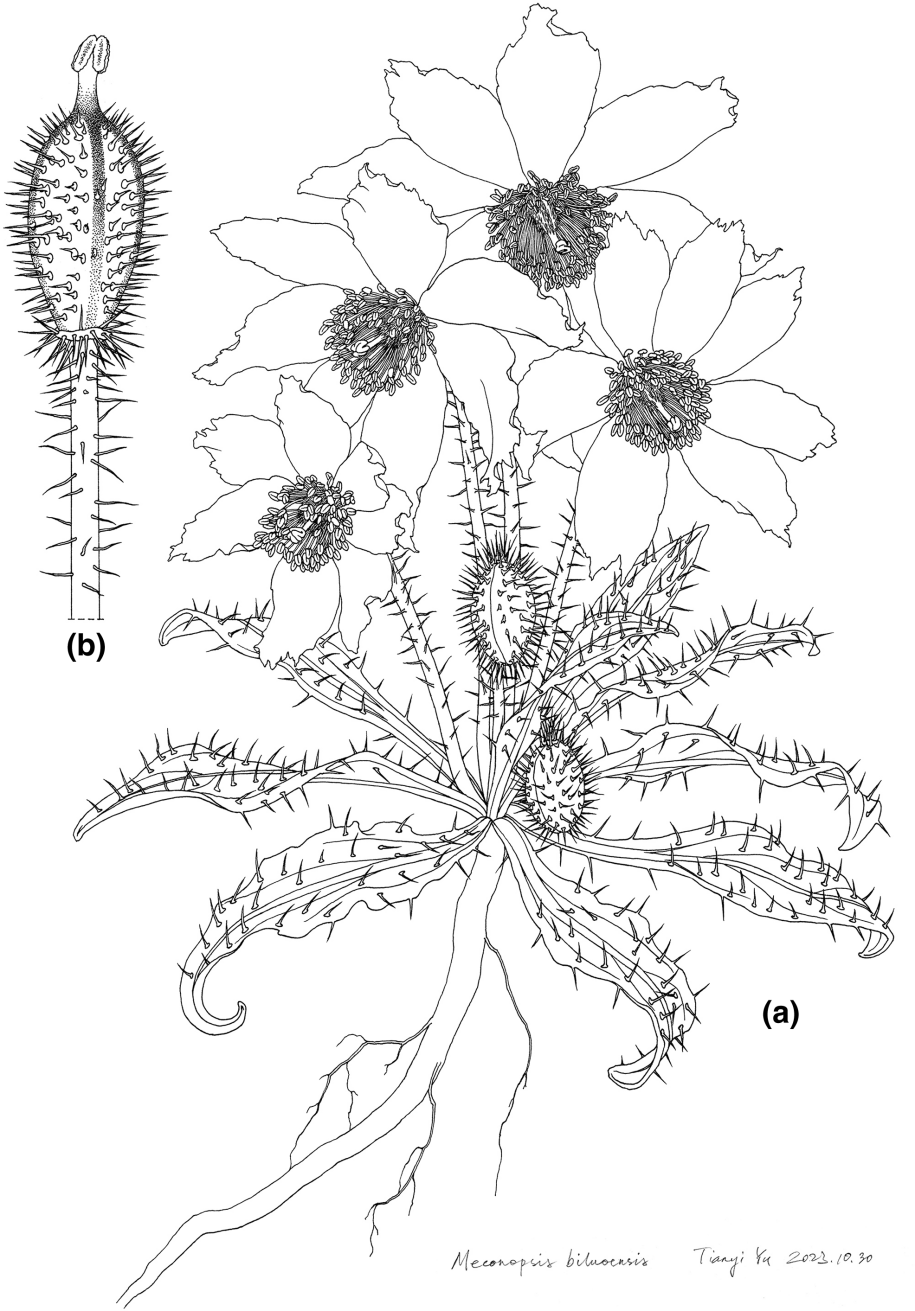
*Meconopsis biluoensis* sp. nov. (a) Habit; (b) Pistil.

*Meconopsis biluoensis* Y. Fan & L. Wei sp. nov. (Figures 7 and 8).

### 4.2 Type

*Holotype*: China, Yunnan, Badi County, Nanjieluo village. 27.9033° N, 98.7930° E, elev. 4200 m, 19, July, 2023, Wangh & Weil 2023012 (Holotype PE!).

*Paratype*: China, Yunnan, Badi County, Nanjieluo village. 27.9161° N, 98.7947° E, elev. 4341 m, 5, August, 2018, Zhouy &Weil 181136 (Paratype BNU!).

## 5 DIAGNOSIS

**FIGURE 9:**
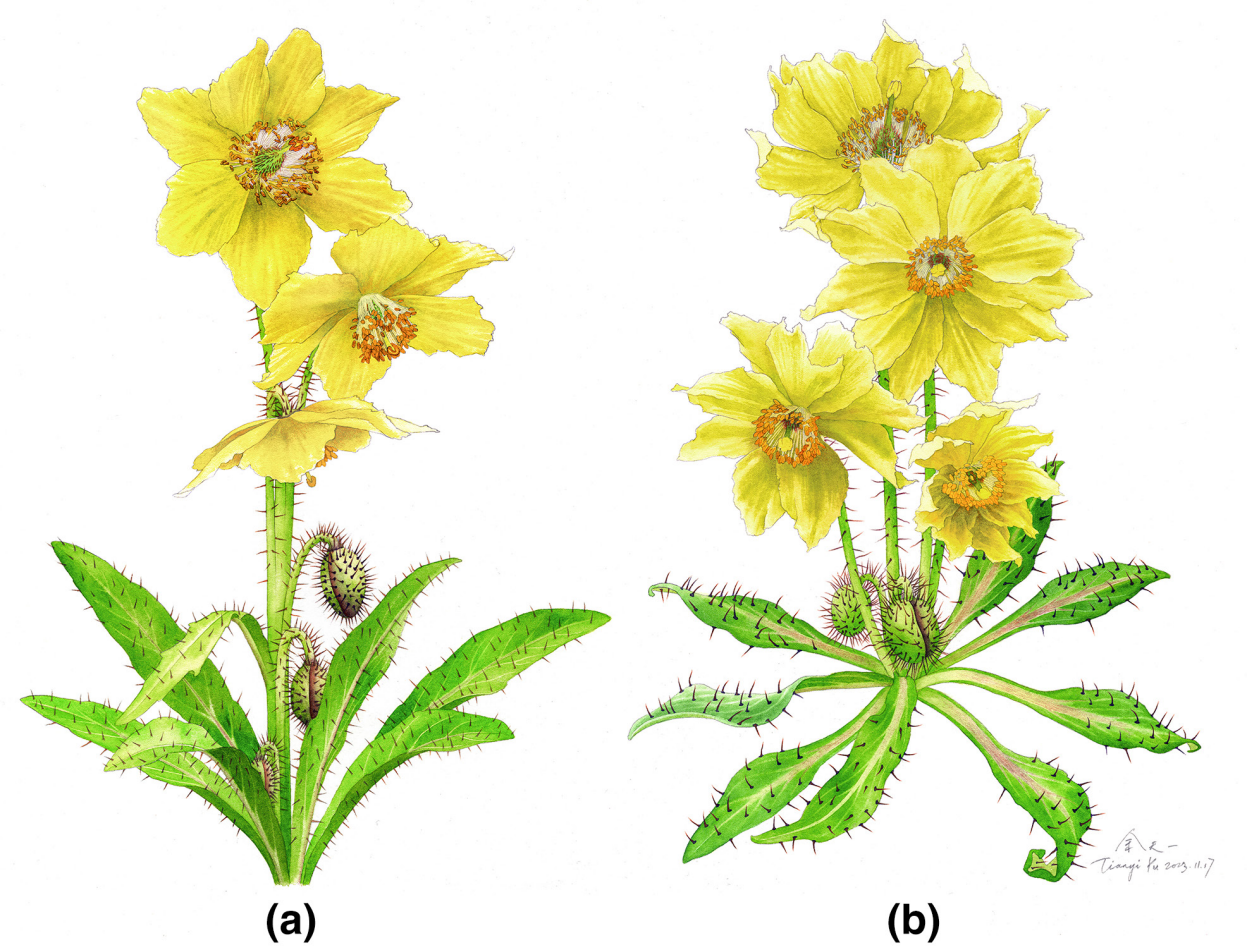
Comparison between *Meconopsis georgei* (a) and *Meconopsis biluoensis* (b), showing the major diagnostic characteristics.

*Meconopsis biluoensis* is morphologically similar to *M. georgei*, but it can be distinguished from the latter in having leafless scapes (vs. lax raceme) and hispid on the leaves with an expanded base (vs. base of hispid scarcely expand). The color of the expanded base is usually brown or black (Figure 9). Additionally, *M. biluoensis* is usually a dwarf plant, rarely exceeding 20 cm, while *M. georgei* is usually taller than 20 cm. Detailed comparisons of diagnostic characteristics are listed in Table 1.

**TABLE 1:** Diagnostic character between *Meconopsis georgei* and *Meconopsis biluoensis*.

Species	Inflorescence	Base of hispid	Height
*M. georgei*	Lax raceme	Scarcely expanded	Usually over 20 cm
*M. biluoensis*	Flowers solitarily born on leafless scapes	Expanded, brown, or black, usually more obvious in mature leaves	Rarely exceeding 20 cm

## 6 DESCRIPTION

Dwarf herb, monocarpic, 10–20 cm tall. Taproot 5–25 cm long, 7–12 mm wide. Stem is usually poorly developed. Leaves all arranged in a lax basal rosette; leaf lamina ovate‐elliptic to lanceolate, 7.0–11.5 × 1.2–2.2 cm, at base attenuate into the petiole, at apex acute to subobtuse, their margin entire to slightly lobed, moderately to densely hirsute with rather sharp patent, pale brown or reddish‐brown bristles, mostly 2–5 mm long with an expanded base; petiole broad‐linear, mostly 2.5–7.0 cm long. Flowers 1–10, borne on the leafless scapes. Pedicel slender, ca 15 cm, sparsely to densely bristly. Sepals 2, adorned with patent bristles. Petals 5–8, primrose‐yellow, rounded, ovate to broad‐elliptic, subacute to subobtuse, along margin often minutely denticulate, otherwise entire. Stamens numerous, with filiform filaments and orange‐yellow anthers. Ovary oblong to ellipsoid, densely covered with stiff, sharp bristles; style 3.5–6.0 mm long; stigma capitate to multi‐lobed. Fruit a capsule, ellipsoid to ellipsoid‐obovoid to cylindrical‐ellipsoid, 15–35 × 6.5–12.0 mm, covered in dense patent to ascending, sharply pointed bristles, 3–5‐valved, splitting for a short distance from the apex at maturity with persistent, relatively short stout, linear, 4–8 mm long style. Seeds falcate‐ellipsoid, irregularly rugose, minutely pitted.

### 6.1 Phenology

Flowering from mid‐July to early August and fruiting from August to September.

### 6.2 Etymology

The species is named after the mountain range to which it was discovered and endemic: Biluo Xueshan Mountain.

### 6.3 Distribution and ecology

This new species is presently only known from the type locality: Nanjieluo village, Badi township, Biluo Xueshan, Weixi, northwest Yunnan, with an elevation range from 4000 to 4200 m. *Meconopsis biluoensis* grows in stony and rocky alpine meadows and slopes, where the environment is often wet and mossy.

### 6.4 Conservation status

During five consecutive years of fieldwork in Biluo Xueshan Mountain, we only found *M. biluoensis* in Nanjieluo. In this area, we found a total of five populations, with each population comprising approximately 10–30 individuals (Wang et al., 2023). Considering that there are areas in Biluo Xueshan Mountain that are difficult to reach, it is also possible that *M. biluoensis* could be distributed in suitable habitats in these regions. However, based on the experience of the current survey, the number of individuals per colony will not exceed 50. Therefore, we estimate the total number of this species to be approximately 500. Based on the above surveys and estimates, we determined its endangerment level to be endangered (EN) based on IUCN (2012), quoting clause C, subclause C.2a.

## AUTHOR CONTRIBUTIONS

**Hao Wang:** Investigation (lead); methodology (lead); writing – original draft (supporting). **Tian‐Yi Yu:** Investigation (supporting); visualization (lead). **Yi Fan:** Investigation (equal); project administration (equal); resources (lead); visualization (supporting). **Lai Wei:** Funding acquisition (lead); project administration (lead); writing – original draft (lead); writing – review and editing (lead).

## FUNDING INFORMATION

This study is supported by Zhilan Foundation, Shenzhen (Grant No. 2022040291B).

## CONFLICT OF INTEREST STATEMENT

There is no conflict of interest to declare.

## Data Availability

All DNA sequences data have been deposited in NCBI, accession numbers: OR797105; OR797104; OR774900; OR774904; OR813927; OR797106; OR797107; OR797108; MK533649.1; MK533646.1; OR774891; OR774892; OR774893; OR774894; OR774895; OR774896; OR774897; OR774898; OR774899; OR774900; OR774901; OR774902; OR774903; OR774904; OR774905; OR774906; OR774907; OR774908; OR805491; OR805492; OR805493; OR805494; OR805495; OR805496; OR805497; OR805498; OR805499; OR805500; OR805501; OR805502; OR805503; OR805504; OR805505; OR805506; OR805507; OR805508; OR805509.

## Appendix

**TABLE A1:** Taxa used in molecular analyses.

Species	DNA type	Self‐sequencing/quote	Accession number	Voucher
*Meconopsis forrestii*	matK gene	Quote	JX087853.1	N/A
*Meconopsis georgei*	matK gene	Quote	JX087856.1	N/A
*Meconopsis lancifolia*	matK gene	Quote	JX087857.1	N/A
*Meconopsis superba*	matK gene	Quote	JX087858.1	N/A
*Meconopsis paniculata*	matK gene	Quote	JX087860.1	N/A
*Meconopsis autumnalis*	matK gene	Quote	JX087861.1	N/A
*Meconopsis wallichii*	matK gene	Quote	JX087863.1	N/A
*Meconopsis quintuplinervia*	matK gene	Quote	JX087865.1	N/A
*Meconopsis delavayi*	matK gene	Quote	JX087866.1	N/A
*Meconopsis florindae*	matK gene	Quote	JX087870.1	N/A
*Meconopsis betonicifolia*	matK gene	Quote	JX087871.1	N/A
*Meconopsis grandi*	matK gene	Quote	JX087873.1	N/A
*Meconopsis torquata*	matK gene	Quote	JX087875.1	N/A
*Meconopsis simplicifolia*	matK gene	Quote	JX087877.1	N/A
*Meconopsis integrifolia*	matK gene	Quote	JX087878.1	N/A
*Papaver alpinum*	matK gene	Quote	JX087879.1	N/A
*Meconopsis primulina*	matK gene	Quote	JX087887.1	N/A
*Meconopsis sinuata*	matK gene	Quote	JX087890.1	N/A
*Meconopsis staintonii*	matK gene	Quote	JX087893.1	N/A
*Meconopsis pseudovenusta*	matK gene	Quote	JX087894.1	N/A
*Meconopsis ganeshensis*	matK gene	Quote	JX087899.1	N/A
*Meconopsis concinna*	matK gene	Quote	JX087902.1	N/A
*Meconopsis chankheliensis*	matK gene	Quote	JX087904.1	N/A
*Meconopsis napaulensis*	matK gene	Quote	JX087906.1	N/A
*Meconopsis aculeata*	matK gene	Quote	JX087912.1	N/A
*Meconopsis dhwojii*	matK gene	Quote	JX087915.1	N/A
*Meconopsis discigera*	matK gene	Quote	JX087918.1	N/A
*Meconopsis bella*	matK gene	Quote	JX087919.1	N/A
*Meconopsis speciosa*	matK gene	Quote	JX087920.1	N/A
*Meconopsis wumungensis*	matK gene	Quote	JX087922.1	N/A
*Meconopsis wilsonii*	matK gene	Quote	JX087924.1	N/A
*Meconopsis gracilipes*	matK gene	Quote	KF494266.1	N/A
*Meconopsis henrici*	matK gene	Quote	KF494267.1	N/A
*Meconopsis zangnanensis*	matK gene	Quote	KF494278.1	N/A
*Meconopsis robusta*	matK gene	Quote	KF777124.1	N/A
*Meconopsis barbiseta*	matK gene	Quote	ON064179.1	N/A
*Meconopsis pinnatifolia*	matK gene	Quote	ON064180.1	N/A
*Meconopsis pratti*	matK gene	Self‐sequencing	OR797105	China, Yunnan: Weixi Xian, Wei L., BNU2023KQ002 (BNU)
*Meconopsis pseudohorridula*	matK gene	Quote	NC061608.1	N/A
*Meconopsis punicea*	matK gene	Quote	MW233592.1	N/A
*Meconopsis horridula*	matK gene	Quote	JX087905.1	N/A
*Meconopsis racemosa*	matK gene	Quote	MK533649.1	N/A
*Meconopsis sulphurea*	matK gene	Self‐sequencing	OR797104	Leaf samples only
*Meconopsis betonicifolia*	matK gene	Quote	OK349678.1	N/A
*Meconopsis biluoensis*	matK gene	Self‐sequencing	OR774900	China, Yunnan: Weixi Xian, Wei L., BNU2023NJ001 (BNU)
*Meconopsis georgei*	matK gene	Self‐sequencing	OR774904	China, Yunnan: Weixi Xian, Wei L., BNU2023XH001 (BNU)
*Meconopsis bijiangensis*	matK gene	Self‐sequencing	OR813927	only leaf sample
*Meconopsis castanea*	matK gene	Self‐sequencing	OR797106	China, Yunnan: Weixi Xian Wei L., BNU2023XH002 (BNU)
*Meconopsis exilis*	matK gene	Self‐sequencing	OR797107	China, Yunnan: Weixi Xian, Wei L., BNU2023XH003 (BNU)
*Meconopsis impedita*	matK gene	Self‐sequencing	OR797108	China, Yunnan: Weixi Xian, Wei L., BNU2023KQ001 (BNU)
*Meconopsis racemosa*	Chloroplast genome	Quote	MK533649.1	N/A
*Meconopsis horridula*	Chloroplast genome	Quote	MK533646.1	N/A
*Meconopsis biluoensis*	Chloroplast genome	Self‐sequencing	OR774891	Leaf samples only
*Meconopsis biluoensis*	Chloroplast genome	Self‐sequencing	OR774892	Leaf samples only
*Meconopsis biluoensis*	Chloroplast genome	Self‐sequencing	OR774893	Leaf samples only
*Meconopsis biluoensis*	Chloroplast genome	Self‐sequencing	OR774894	Leaf samples only
*Meconopsis biluoensis*	Chloroplast genome	Self‐sequencing	OR774895	Leaf samples only
*Meconopsis biluoensis*	Chloroplast genome	Self‐sequencing	OR774896	Leaf samples only
*Meconopsis biluoensis*	Chloroplast genome	Self‐sequencing	OR774897	Leaf samples only
*Meconopsis biluoensis*	Chloroplast genome	Self‐sequencing	OR774898	Leaf samples only
*Meconopsis biluoensis*	Chloroplast genome	Self‐sequencing	OR774899	Leaf samples only
*Meconopsis biluoensis*	Chloroplast genome	Self‐sequencing	OR774900	Leaf samples only
*Meconopsis biluoensis*	Chloroplast genome	Self‐sequencing	OR774901	Leaf samples only
*Meconopsis biluoensis*	Chloroplast genome	Self‐sequencing	OR774902	Leaf samples only
*Meconopsis biluoensis*	Chloroplast genome	Self‐sequencing	OR774903	Leaf samples only
*Meconopsis georgei*	Chloroplast genome	Self‐sequencing	OR774904	Leaf samples only
*Meconopsis georgei*	Chloroplast genome	Self‐sequencing	OR774905	Leaf samples only
*Meconopsis georgei*	Chloroplast genome	Self‐sequencing	OR774906	Leaf samples only
*Meconopsis georgei*	Chloroplast genome	Self‐sequencing	OR774907	Leaf samples only
*Meconopsis georgei*	Chloroplast genome	Self‐sequencing	OR774908	Leaf samples only
*Meconopsis biluoensis*	Ribosomal RNA gene	Self‐sequencing	OR805491	Leaf samples only
*Meconopsis biluoensis*	Ribosomal RNA gene	Self‐sequencing	OR805492	Leaf samples only
*Meconopsis biluoensis*	Ribosomal RNA gene	Self‐sequencing	OR805493	Leaf samples only
*Meconopsis biluoensis*	Ribosomal RNA gene	Self‐sequencing	OR805494	Leaf samples only
*Meconopsis biluoensis*	Ribosomal RNA gene	Self‐sequencing	OR805495	Leaf samples only
*Meconopsis biluoensis*	Ribosomal RNA gene	Self‐sequencing	OR805496	Leaf samples only
*Meconopsis biluoensis*	Ribosomal RNA gene	Self‐sequencing	OR805497	Leaf samples only
*Meconopsis biluoensis*	Ribosomal RNA gene	Self‐sequencing	OR805498	Leaf samples only
*Meconopsis biluoensis*	Ribosomal RNA gene	Self‐sequencing	OR805499	Leaf samples only
*Meconopsis biluoensis*	Ribosomal RNA gene	Self‐sequencing	OR805500	Leaf samples only
*Meconopsis biluoensis*	Ribosomal RNA gene	Self‐sequencing	OR805501	Leaf samples only
*Meconopsis biluoensis*	Ribosomal RNA gene	Self‐sequencing	OR805502	Leaf samples only
*Meconopsis bijiangensis*	Ribosomal RNA gene	Self‐sequencing	OR805503	Leaf samples only
*Meconopsis georgei*	Ribosomal RNA gene	Self‐sequencing	OR805504	Leaf samples only
*Meconopsis georgei*	Ribosomal RNA gene	Self‐sequencing	OR805505	Leaf samples only
*Meconopsis georgei*	Ribosomal RNA gene	Self‐sequencing	OR805506	Leaf samples only
*Meconopsis georgei*	Ribosomal RNA gene	Self‐sequencing	OR805507	Leaf samples only
*Meconopsis georgei*	Ribosomal RNA gene	Self‐sequencing	OR805508	Leaf samples only
*Meconopsis biluoensis*	Ribosomal RNA gene	Self‐sequencing	OR805509	Leaf samples only
